# Craniectomy versus craniotomy: What can we do for acute subdural hematoma?

**DOI:** 10.1002/agm2.12322

**Published:** 2024-06-14

**Authors:** Shuo Zhang, Guoyi Gao, Weiming Liu

**Affiliations:** ^1^ Department of Neurosurgery Beijing Tiantan Hospital, Capital Medical University Beijing China

**Keywords:** acute subdural hematoma, craniectomy, craniotomy, intracranial pressure

## Abstract

Compared with hematoma evacuation craniotomy, decompressive craniectomy has a higher incidence of intracranial complications and no outcome benefit over craniotomy, which gives surgeons a safer decision‐making options during surgery.

## 1 BACKGROUND

Acute subdural hematoma (ASDH) is one of the common complications after traumatic brain injury (TBI) that warrants surgical evacuation. It often progresses rapidly and has a poor clinical prognosis, due to the mass effect caused by the hematoma and the invasion of the adjacent cerebral cortex and parenchyma. Although with the advancement of surgical techniques, the survival rate after ASDH increased from 59% in 1994–1998 to 73% in 2009–2013,^1^ the surgical indications for hematoma evacuation and whether to perform decompression surgery are still controversial which depends on the patient's level of consciousness, pupil status, neuroimaging findings, and intracranial pressure.^2^ Hematoma evacuation is required in a quarter of patients to save lives.^3^, ^4^, ^5^ At the same time, craniectomy was performed in another 25% of these patients to prevent postoperative brain edema or intraoperative brain tissue swelling, this proportion can be as high as nearly 50% in patients with severe traumatic brain injury in China.^6^ In most cases, the primary motivation for emergency hematoma removal is to save lives or the noticeable space‐occupying effect on computed tomography (CT) images,^4^ however, if the intracranial pressure during emergency surgery allows, whether to remove the bone flap is an essential factor that probably affect the prognosis of patients.

## 2 CURRENT STRATEGIES

Recently, in the New England Journal of Medicine, P. J. Hutchinson and colleagues address the comparison of surgical outcomes of craniotomy and decompressive craniectomy (RESCUE‐ASDH trial),^7^ it is an investigator‐initiated, international, multicenter, pragmatic, randomized trial of patent with a bone flap greater than 11 cm in diameter who accepted the hematoma evacuation. Extended Glasgow Outcome Scale (GOSE) was used to evaluate patients’ outcomes after surgery 12 months later. The mortality and prognosis of the decompressive craniectomy and craniotomy groups were compared at 1, 6 months, and 1 year after operation, and there was no significance in disability and quality‐of‐life outcomes between two groups. However, the craniotomy group may have to face re‐surgery due to difficult‐to‐control brain edema, while the decompressive craniectomy group may face more trauma‐related complications.

The key conclusions of RESCUE‐ASDH trial provide an essential reference for the decision‐making of whether to remove the bone flap during the subdural hematoma surgery, that is, if no encephalocele occurs immediately during the operation, reduction of the skull with brain tissue no higher than the plane level of the skull does not increase the risk of poor prognosis. The conclusions are consistent with the recent findings of Thomas A. van Essen et al.,^5^ which pose a significant challenge to previous clinical practice.

Compared with intracranial pressure monitoring in TBI patients, imaging and clinical examination have been considered to be more valuable for clinical decision‐making, and the necessity of invasive intracranial pressure monitoring is still controversial.^8^ However, the recent SYNAPSE‐ICU study by Chiara Robba et al. reemphasized the necessity for intracranial pressure monitoring, especially for patients with severe TBI, it can reduce the mortality of patients after hemorrhage to some extent.^9^ We believe that in patients undergoing emergency surgical evacuation of hematoma, invasive intracranial pressure monitoring after surgery is crucial for nondecompressive craniectomy patients to detect potential cerebral edema as well as increased intracranial pressure due to hemorrhage events as early as possible. Because decompressive craniectomy was performed completely randomized in this study, although there was no difference in quality of life between two groups, there was a higher rate of secondary surgery in the craniotomy group. If combined with intraoperative brain tissue pulsations, transcranial Doppler monitoring, or preoperative CT perfusion measurement, potential cerebral ischemic events can be recognized more timely, decompressive craniectomy probably becomes a safer choice and even avoids secondary surgery.

In addition, economic burden is also recognized as a crucial factor affecting clinical treatment, especially in underdeveloped regions. The costs of cranioplasty, hydrocephalus treatment, and prolonged anti‐infective therapy after decompressive craniectomy may be prohibitive for patients.

## 3 ACUTE SUBDURAL HEMATOMA IN THE ELDERLY

The survival rate of ASDH in the elderly (>65 years old) is significantly lower than that of the young population. Studies have shown that the mortality rate of ASDH is up to 40%,^10^, ^11^ and only 1/8 of patients with severe craniocerebral trauma can fully recover self‐care ability within 6 months after injury.^12^ A considerable proportion of older adults routinely take anticoagulant drugs and antiplatelet drugs, which increases the probability of intracranial hemorrhage after trauma to 15.9%.^13^ It is challenging to solve the occupying effect of ASDH through burr hole drainage, and serious systemic complications often occur in elderly patients after craniotomy, leading to poor prognosis of surgery. A considerable number of elderly patients are in serious condition with other severe comorbidities, which significantly increase the incidence of surgery‐related complications and even make surgical treatment impossible.^7^ The latest review also mentioned that it is difficult for elderly patients (age >70 years) to benefit from emergency craniotomy. A better choice may be to wait for 2 weeks to make the hematoma chronic and perform drilling and drainage under the condition of critical monitoring and necessary symptomatic treatment.^14^ It seems that brain atrophy in the elderly population provides some space for potential brain swelling. However, there are no RCT studies in the elderly population associated with ASDH surgery to confirm the benefit between craniotomy and decompressive craniotomy. The potential impact on prognosis due to wound complications and surgical‐site infections was also mentioned in the findings of the RESCUE‐ASDH trial.^7^ A craniotomy can be a second attack of severe neurotrauma, and its associated complications can be fatal, making it more difficult to compare the long‐term outcomes after craniotomy and decompressive craniectomy in older people.

## 4 CONCLUSION

The surgical indication of ASDH should be comprehensively judged based on the patient's consciousness state and CT image data. Compared with hematoma evacuation craniotomy, decompressive craniectomy has a higher incidence of intracranial complications and no outcome benefit over craniotomy, which gives surgeons safer decision‐making options during surgery. For severe TBI patients, the need for intracranial pressure monitoring during emergency craniotomy remains to be carefully evaluated.

## AUTHOR CONTRIBUTIONS

Weiming Liu conceptualized the manuscript. Shuo Zhang did the literature search and wrote the manuscript draft. Guoyi Gao critically revised the manuscript draft.

## FUNDING INFORMATION

This work was not supported by any source.

## CONFLICT OF INTEREST STATEMENT

The authors declare that they have no conflict of interest.
